# ﻿*Chimena* gen. nov., a new spider genus (Araneae, Mysmenidae) from China, with descriptions of two new species and a new combination

**DOI:** 10.3897/zookeys.1125.85741

**Published:** 2022-10-20

**Authors:** Yucheng Lin, Shuqiang Li

**Affiliations:** 1 Key Laboratory of Bio-resources and Eco-environment (Ministry of Education), College of Life Sciences, Sichuan University, Chengdu 610065, China; 2 The Sichuan key Laboratory for Conversation Biology of Endangered Wildlife, Sichuan University, Chengdu 610065, China; 3 Institute of Zoology, Chinese Academy of Sciences, Beijing 100101, China

**Keywords:** Diagnosis, Hainan, mysmenids, new genus, symphytognathoids, Taiwan, taxonomy

## Abstract

A new mysmenid genus, *Chimena***gen. nov.**, is reported from China. Two new species: *C.qiong***sp. nov.** (Hainan, ♂♀, the type species) and *C.nantou***sp. nov.** (Taiwan, ♀) are illustrated and described in detail. A new combination is suggested: *Chimenataiwanica* (Ono, 2007) **comb. nov.** (Taiwan, ♂♀, transferred from *Mysmena* Simon, 1894). The molecular phylogeny and morphological characters were used to discuss the taxonomy and circumscription of the newly erected genus.

## ﻿Introduction

The spider family Mysmenidae Petrunkevitch, 1928 includes 158 extant species in 14 genera (WSC 2022), making it the second most species-rich spider family of the symphytognathoids. Known species of Mysmenidae are recorded mainly in Asia and South America (Brescovit and Lopardo 2008; Lin and Li 2008, 2013, 2014, 2016; Miller et al. 2009; Feng et al. 2019; Li and Lin 2019; Dupérré and Tapia 2020). Lopardo et al. (2011) suggested that this family is distributed worldwide, and its diversity is grossly underestimated due to their small size and cryptic lifestyle.

In Asia, nearly 50 species of nine genera have been recorded. Simon (1895a, b) first reported three species from Sri Lanka and the Philippines. Baert (1988) described three species from Sulawesi, Indonesia. The Vietnamese mysmenids were first reported by Lin and Li (2014), and three species were recorded. Nearly 40 species from South China have been described in the past 20 years, more than half of them from Yunnan Province (Lin and Li 2008, 2013; Miller et al. 2009). However, the extraordinary species diversity of Mysmenidae in China and surrounding areas needs to be further investigated.

The aim of this paper is to expand the knowledge about the species diversity of Chinese mysmenid spiders by describing a new genus and two new species and proposing one new combination.

## ﻿Materials and methods

### ﻿Material

The mysmenid specimens in this study were collected in Taiwan and Hainan, China, between June 2011 and July 2013. All the specimens were collected by sifting leaf litter or by hand and stored in 95% ethanol at –20 °C.

### ﻿Molecular data

We selected seven specimens from two new species and used the prosoma and all of the legs to extract genomic DNA to amplify COI, H3, 16S, 18S, and 28S. DNA was extracted with the TIANamp Micro DNA Kit (**TIANGEN**) following the manufacturer’s protocol for animal tissues. The five gene fragments were amplified in 25μL reactions. Primer pairs and PCR protocols are given in Table 1. Raw sequences were edited and assembled using BioEdit v.7.2.5 (Hall 1999). New sequences from this study were deposited in GenBank, and the accession numbers are reported in Table 2. All molecular vouchers and material are stored in the Natural History Museum of Sichuan University in Chengdu (**NHMSU**), China.

**Table 1.** The loci, primer pairs, and PCR protocols used in this study.

Locus	Annealing temperature/time	Direction	Primer	Sequence 5’→3’	Reference
16S	46.45°/30s	F	16sb2_12864	CTCCGGTTTGAACTCAGATCA	Hormiga et al. 2003
		R	LR-J-13360	GTAAGGCCTGCTCAATGA	Feng et al. 2019
	47°/30s	F	16S-A	CGCCTGTTTATCAAAAACAT	Palumbi et al. 1991
		R	16S-B	CTCCGGTTTGAACTCAGATCA	
18S	52.1°/30s	F	18s_1F	TACCTGGTTGATCCTGCCAGTAG	Giribet et al. 1996
		R	18s_1000R	GTGGTGCCCTTCCGTCAATT	Balczun et al. 2005
28SD2	54.9°/30s	F	28sa	GACCCGTCTTGAAACACGGA	Rix et al. 2008
		R	LSUR	GCTACTACCACCAAGATCTGCA	
COI	48.95°/30s	F	LCO1490	GGTCAACAAATCATAAAGATATTGG	Folmer et al. 1994
		R	HCO2198	TAAACTTCAGGGTGACCAAAAAATCA	
	46°/30s	F	LCO1490	GGTCAACAAATCATAAAGATATTGG	Simon et al. 1994
		R	COI-Nancy	CCCGGTAAAATTAAAATATAAACTTC	
H3	48°/30s	F	H3af	ATGGCTCGTACCAAGCAGACVGC	Colgan et al. 1998
		R	H3ar	ATATCCTTRGGCATRATRGTGAC	
	50°/30s	F	H3nf	ATGGCTCGTACCAAGCAGAC	
		R	H3nr	ATRTCCTTGGGCATGATTGTTAC	

**Table 2.** GenBank accession numbers for newly generated DNA sequences.

Species	Identifier	16S	18S	28S	COI	H3
*Chimenataiwanica*	TW02	OP022513	OP022536	OP022496	OP053348	OP095882
*Chimenaqiong* sp. nov.	HN01	OP022508	OP022530	OP022484	OP021152	OP095876
	HN02	OP022509	OP022531	OP022485	OP053341	OP095877
	HN05	OP022510	OP022532	OP022486	OP053342	OP095878
	HN08	OP022511	OP022533	OP022487	–	OP095879
	HN09	OP022512	OP022535	OP022495	OP053347	OP095881
	HN10	–	OP022534	OP022488	–	OP095880
*Chaneasuukyii*	GlgMY01	OP022523	OP022541	OP022501	OP053353	OP095887
	GlgMY02	OP022524	OP022542	OP022502	OP053354	OP095888
	GlgMY03	OP022525	OP022543	OP022503	OP053355	OP095889
	GlgMY04	OP022526	OP022544	OP022504	OP053356	OP095890
	GlgMY05	OP022527	OP022545	OP022505	OP053357	OP095891
	GlgMY80	OP022528	OP022546	OP022506	OP053358	OP095892
	GlgMY98	OP022529	OP022547	OP022507	OP053359	OP095893
*Chaneavoluta*	XZ01	OP022516	OP022548	OP022489	OP023992	OP095896
	XZ02	OP022517	OP022549	OP022490	OP053345	OP095897
	XZ03	OP022518	OP022550	OP022491	OP053346	OP095898
	XZ04	OP022519	OP022551	OP022492	OP038899	OP095899
*Chanea* sp.	MS_261_MYA	OP022522	OP022539	OP022499	OP053351	OP095885
*Chanea* sp.	MS_263_MYA	–	OP022540	OP022500	OP053352	OP095886
*Chanea* sp.	MS_250_INN	OP022520	OP022537	OP022497	OP053349	OP095883
*Chanea* sp.	MS_251_INN	OP022521	OP022538	OP022498	OP053350	OP095884
*Chanea* sp.	INNE02	OP022514	OP022552	OP022493	OP053343	OP095894
*Chanea* sp.	INNE03	OP022515	OP022553	OP022494	OP053344	OP095895

We analysed data from 50 species of symphytognathoids including members of the families Theridiosomatidae Simon, 1881, Mysmenidae, Anapidae Simon, 1895, and Symphytognathidae Hickman, 1931. We used the MAFFT v.7.450 online server (https://mafft.cbrc.jp/alignment/server/) with default parameters to align the sequences of *Chimena* and *Chanea* species involved in this study. All sequences were concatenated in SequenceMatrix v.1.7.8 (Vaidya et al. 2011). PartitionFinder2 (Lanfear et al. 2017) was used to identify the best-fit models of molecular evolution for each locus. GTR+I+G was selected for COI, H3, 18S, and 28S, and GTR+G was selected for 16S.

We analysed the data using both maximum parsimony (MP) and Bayesian Inference (BI). The MP tree was constructed using MEGA X (Kumar et al. 2018) with TBR (Tree-Bisection-Reconnection) branch swapping and 2000 bootstrap replicates with all other parameters set to default. BI was performed using MrBayes v.3.2.7 (Ronquist et al. 2012) on the Cipres Science Gateway (Miller et al. 2010), with four Markov Chains (MCMCs) with default heating parameters for 50,000,000 generations until the average standard deviation of split frequencies was less than 0.01. The Markov chains were sampled every 1000 generations, and the first 25% of sampled trees were burn-in.

### ﻿Morphological data

Specimens were examined and measured under a Leica M205 C stereomicroscope. Further details were examined using an Olympus BX51 compound microscope. Male palps and epigynes were examined and photographed after dissection. They were treated in lactic acid for several minutes, and subsequently embedded in Hoyer’s Solution before photographing. Photos were made with a Canon EOS 60D wide zoom digital camera (8.5 megapixels) mounted on the Olympus BX51 compound microscope. Images were combined using Helicon Focus v.3.10 software (Khmelik et al. 2006). All measurements are in millimetres. Leg measurements are given as follows: total length (femur, patella, tibia, metatarsus, and tarsus). Abbreviations of institutions and morphological terminology are given in Table 3. References to figures in cited papers are listed in lowercase (fig. or figs), and figures in this paper are noted with an initial capital (Fig. or Figs).

**Table 3.** List of abbreviations used in the text or figures.

Morphological terminologies			
**AER**	anterior eye row	**FD**	fertilization ducts
**ALE**	anterior lateral eyes	**MN**	male metatarsal nodule at distal-prolaterally
**AME**	anterior median eyes	**MS**	male metatarsal clasping spine
**BH**	basal haematodocha	**PC**	paracymbium
**CD**	copulatory ducts	**PER**	posterior eye row
**CS**	cheliceral spines rooted at base	**PLE**	posterior lateral eyes
**CyC**	cymbial conductor	**PME**	posterior median eyes
**CyF**	cymbial fold	**S**	spermathecae
**CyFs**	setae on cymbial fold	**SD**	spermatic duct
**CyP1**	process on cymbial conductor	**SP**	scape
**CyP2**	process on paracymbium	**St**	subtegulum
**E**	embolus	**Ti**	palpal tibia
**Institutions**			
**FRIT**	Forestry Research Institute of Taipei, Taipei, China		
**IZCAS**	Institute of Zoology, Chinese Academy of Sciences, Beijing, China		
**NSMT**	Department of Zoology, National Science Museum, Tokyo, Japan		
**NHMSU**	Natural History Museum of Sichuan University, Chengdu, China		

## ﻿Results

### ﻿Phylogenetic analysis

**Figure 1.**
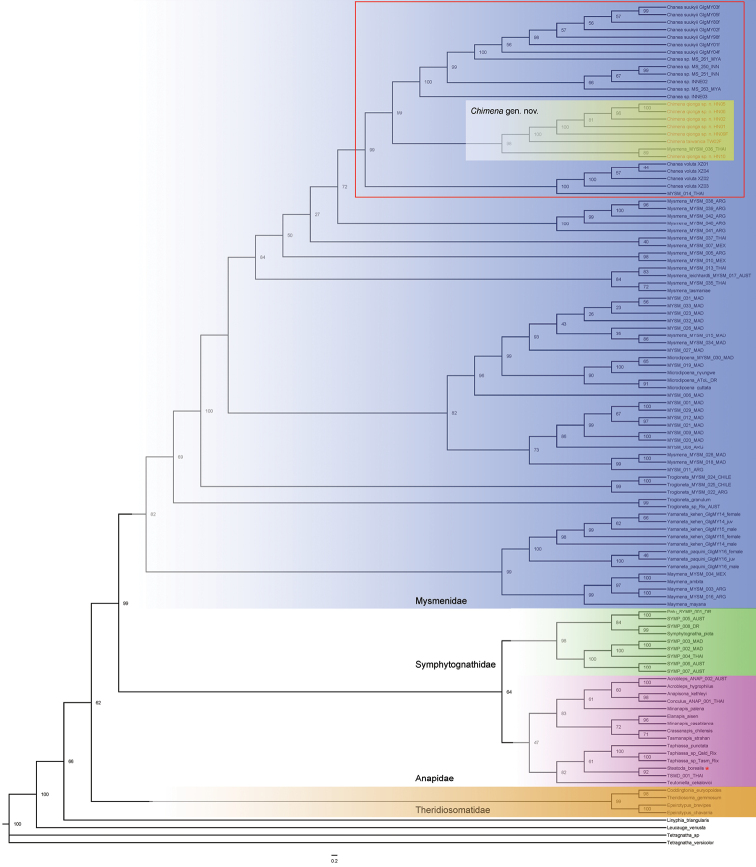
Tree topology obtained by maximum likelihood. Numbers at nodes are bootstrap values. Тhe clade of *Chimena* gen. nov. (yellow) + *Chanea* is nested within Mysmenidae (blue). Further clades are Symphytognathidae (green), Anapidae (pink) and Theridiosomatidae (orange).

**Figure 2.**
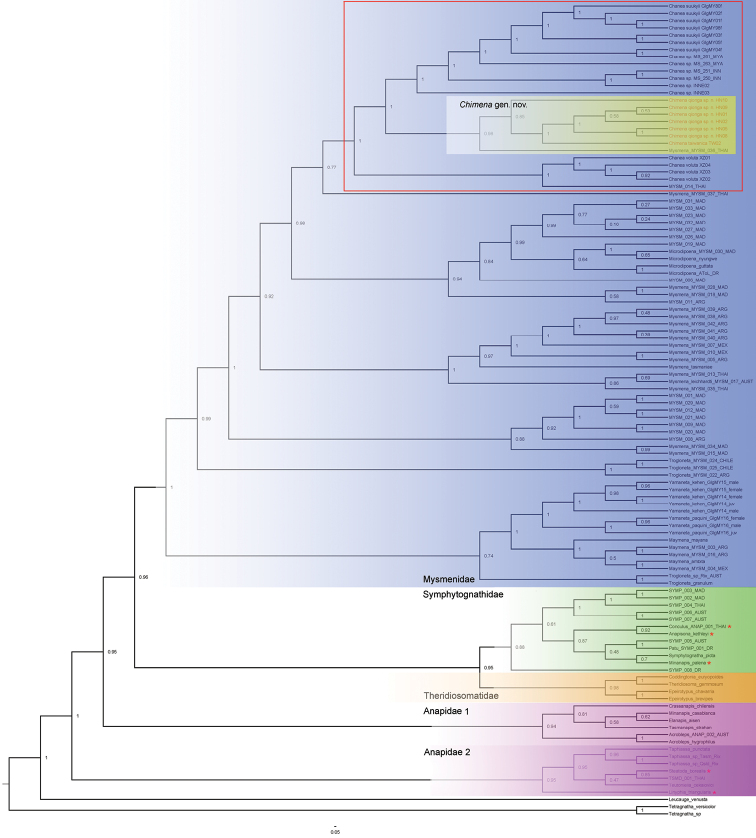
Bayesian inference tree. Numbers at nodes posterior probabilities. The monophyly of Mysmenidae (blue), Theridiosomatidae (orange), and *Chimena* gen. nov. (yellow) are highly supported. Note the paraphyly of Anapidae (pink) and placement of *Steatoda_borealis* and *Linyphia_triangularis* within “Anapidae 2”; three anapid species (red star) are nested within Symphytognathidae (green).

The topologies from both the MP and BI analyses (Figs 1, 2) showed mysmenids and theridiosomatids were highly supported as monophyletic in both analyses, although the position of theridiosomatids was not consistent between analyses. Symphytognathidae was rendered polyphyletic by three anapid species. The monophyly of Anapidae is not strongly supported and is rendered paraphyletic due to the placement of the theridiid *Steatodaborealis* (Hentz, 1850). In the BI tree anapids are divided into two highly supported clades that we refer to as “Anapidae 1” and “Anapidae 2” (Fig. 2), but Anapidae 2 is rendered polyphyletic by the theridiid *Steatodaborealis* and the linyphiid *Linyphiatriangularis* (Clerck, 1757).

### ﻿Taxonomy

#### Mysmenidae Petrunkevitch, 1928

##### ﻿ Chimena gen. nov.

Taxon classificationAnimaliaAraneaeMysmenidae

7DC578FA-3E86-5796-8B3B-CDD4387AE989

https://zoobank.org/76A8EC9A-6199-4D97-810E-966B5C675A56

###### Type species.

*Chimenaqiong* sp. nov.

###### Etymology.

The generic name is a combination of the first three letters of China and the latter half of *Mysmena*. The gender is feminine.

###### Diagnosis.

**Figure 3.**
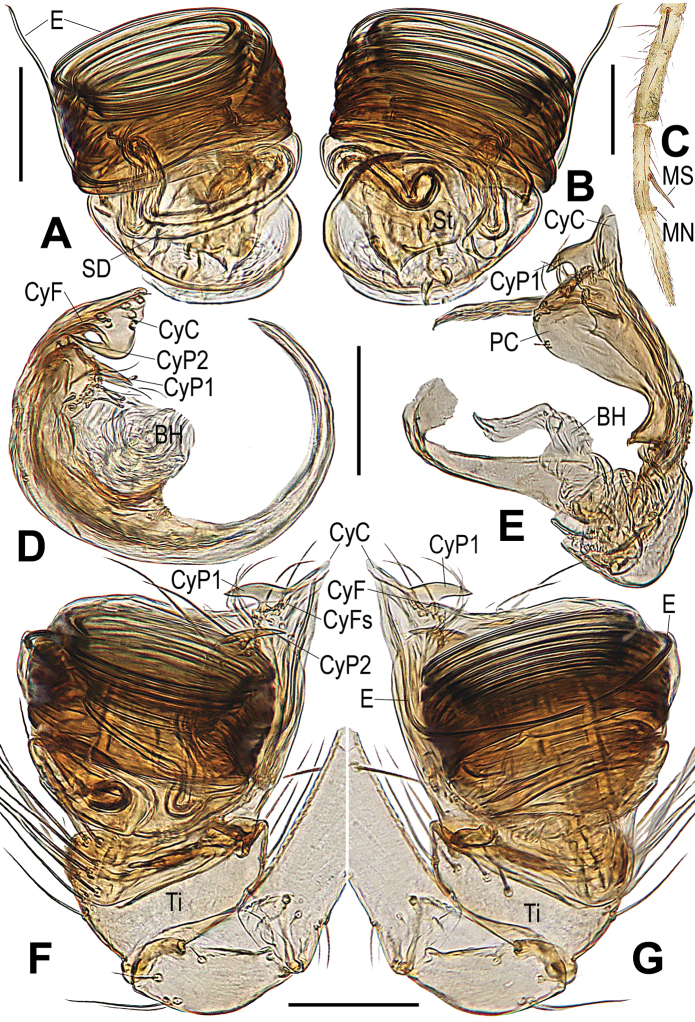
*Chimenaqiong* sp. nov., male **A** left palpal bulb, retrolateral **B** palpal bulb, prolateral **C** distal segments of right leg I, prolateral **D** cymbium, apical **E** cymbium, retrolateral **F** left palp, retrolateral **G** left palp, prolateral. Abbreviations: BH basal haematodocha; CyC cymbial conductor; CyF cymbial fold; CyFs setae on cymbial fold; CyP1 process on cymbial conductor; CyP2 process on paracymbium; PC paracymbium; E embolus; MN male metatarsal nodule at distal-prolaterally; MS male metatarsal clasping spine; SD spermatic duct; St subtegulum; Ti palpal tibia. Scale bars: 0.10 mm.

**Figure 4.**
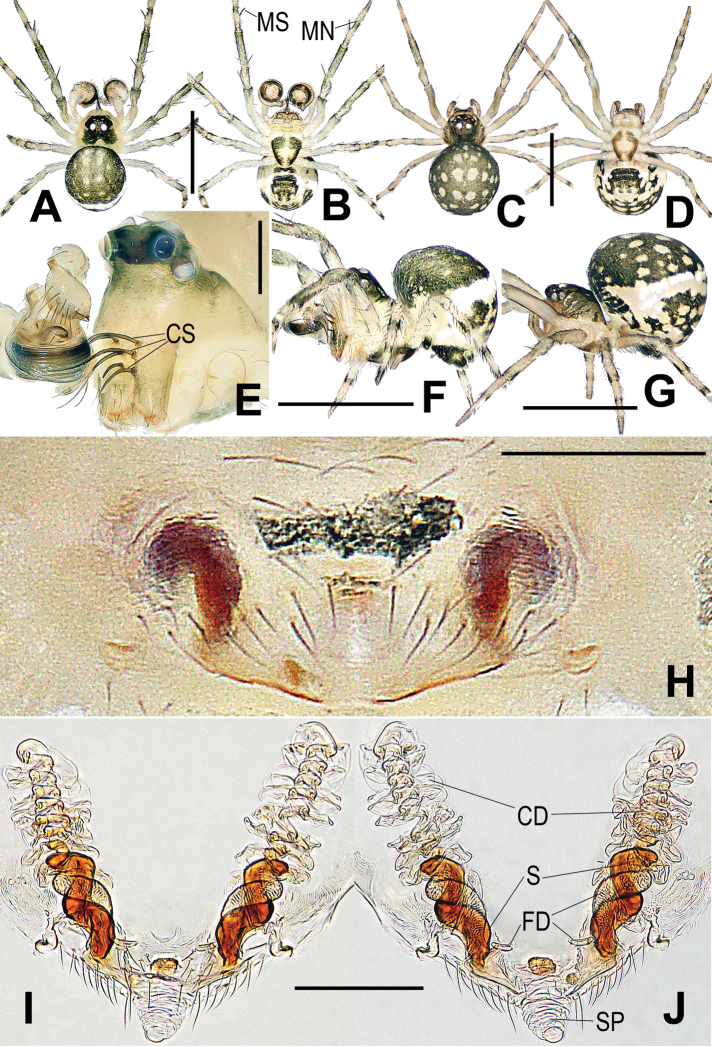
*Chimenaqiong* sp. nov., male (**A, B, E, F**) and female (**C, D, G–J**) **A, C** habitus, dorsal **B, D** habitus, ventral **E** prosoma, front-lateral, **F, G** habitus, lateral **H** epigyne, ventral **I** vulva, ventral **J** vulva, dorsal. Abbreviations: CD copulatory ducts; CS cheliceral spines rooted at base; FD fertilization ducts; MN male metatarsal nodule at distal-prolaterally; MS male metatarsal clasping spine; S spermathecae; SP scape. Scale bars: 0.50 mm (**A–D, F, G**); 0.20 mm (**E**); 0.10 mm (**H–J**).

**Figure 5.**
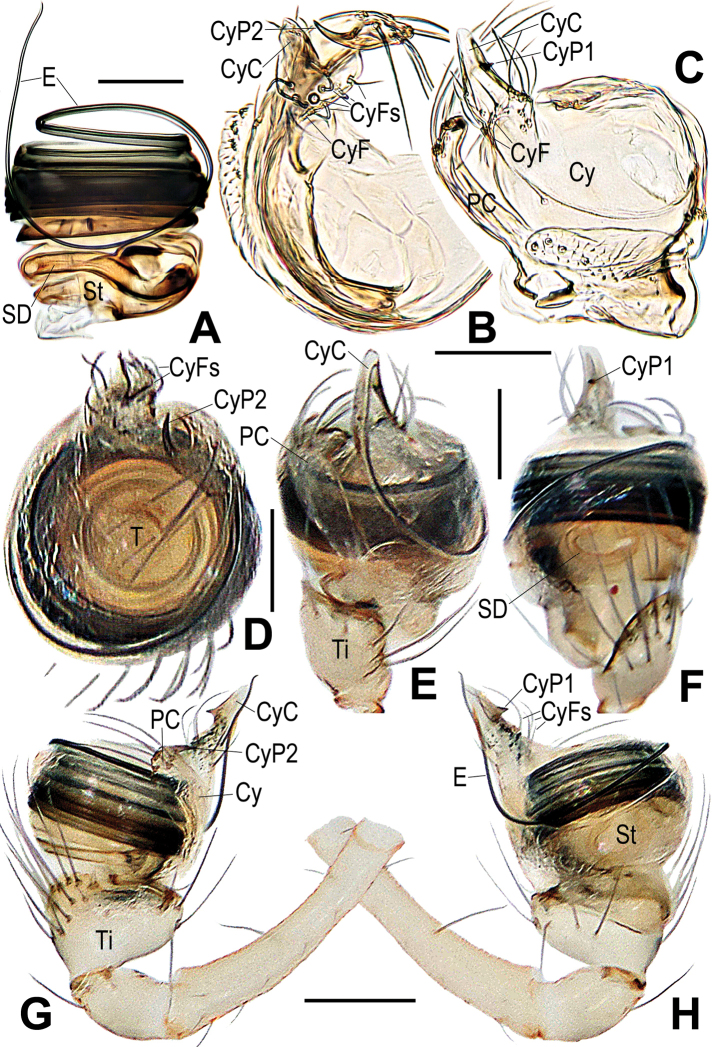
*Chimenataiwanica* (Ono, 2007) comb. nov., male **A** left palpal bulb, retrolateral **B** cymbium, apical **C** cymbium, dorsal-retrolateral **D** left palp, apical **E** left palp, ventral **F** left palp, dorsal **G** left palp, retrolateral **H** left palp, retrolateral. Abbreviations: Cy cymbium; CyC cymbial conductor; CyF cymbial fold; CyFs setae on cymbial fold; CT cymbial tooth; CyP2 process on paracymbium; PC paracymbium; E embolus; SD spermatic duct; St subtegulum; T tegulum; Ti palpal tibia. Scale bars: 0.10 mm.

**Figure 6.**
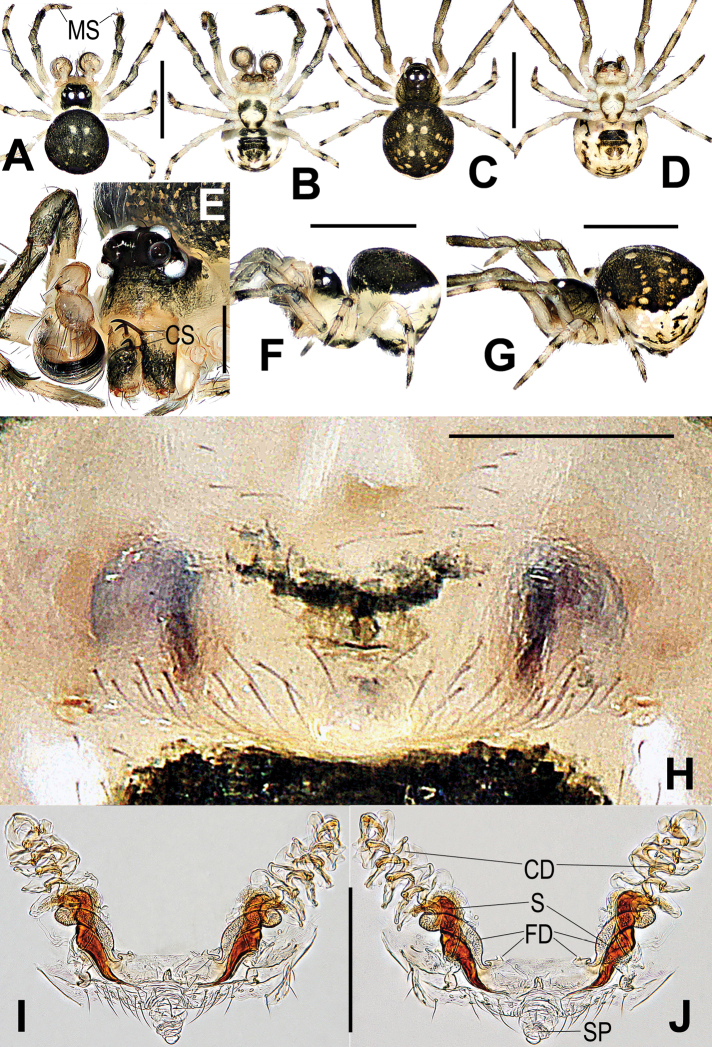
*Chimenataiwanica* (Ono, 2007) comb. nov., male (**A, B, E, F**) and female (**C, D, G–J**) **A, C** habitus, dorsal **B, D** habitus, ventral **E** prosoma, front-lateral **F, G** habitus, lateral **H** epigyne, ventral **I** vulva, ventral **J** vulva, dorsal. Abbreviations: CD copulatory ducts; CS cheliceral spines rooted at base; FD fertilization ducts; MS male metatarsal clasping spine; S spermathecae; SP scape. Scale bars: 0.50 mm (**A–D, F, G**); 0.20 mm (**E**); 0.10 mm (**H–J**).

**Figure 7.**
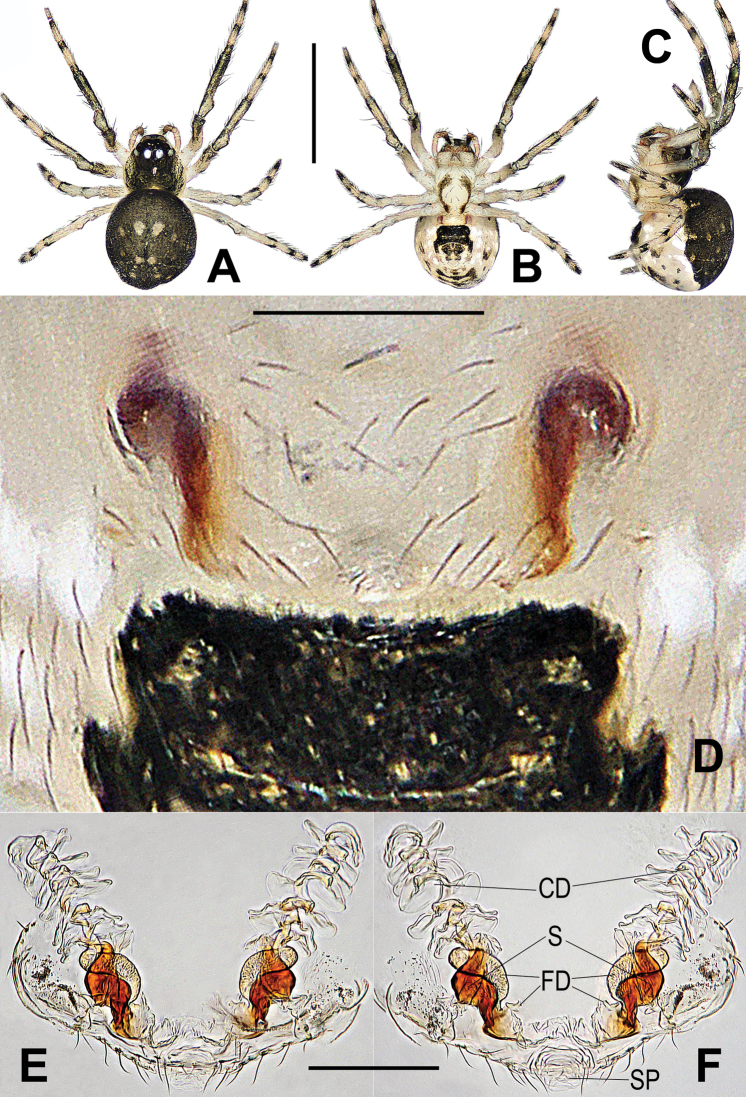
*Chimenanantou* sp. nov., holotype female **A** habitus, dorsal **B** habitus, ventral **C** habitus, lateral **D** epigyne, ventral **E** vulva, ventral **F** vulva, dorsal. Abbreviations: CD copulatory ducts; FD fertilization ducts; S spermathecae; SP scape. Scale bars: 0.50 mm (**A–C**); 0.10 mm (**D–F**).

*Chimena* gen. nov. differs from other mysmenid genera by the presence of strong spines on the chelicerae of males (as in some Chinese species of *Gaoligonga* Miller, Griswold & Yin, 2009 and *Mysmena* Miller, Griswold & Yin, 2009; see fig. 38A in Miller et al. 2009, fig. 8C in Lin and Li 2014, and figs 5E, 6A, 7E in Lin and Li 2008); a very long embolus spiralling around the bulb at least 5 times; and the spermathecae near the posterior margin of the epigyne; the copulatory ducts are highly coiled and extend anteriorly. *Chimena* gen. nov. is morphologically similar to *Chanea* Miller, Griswold & Yin, 2009 in having an extremely coiled embolus (cf. Figs 3A, 5A; figs 49A–B, 51A–B in Miller et al. 2009; figs 3A in Lin and Li 2016) and a membranous, translucent, wrinkled scape (Figs 4J, 6J, 7F; fig. 4D in Lin and Li 2016). Males can be distinguished by the presence of a cymbial process (CyP1, CyP2), which is absent in *Chanea* (Figs 3D, 3F, 5B, 5G vs. 49A, 49B in Miller et al. 2009 and figs 2C, 3C in Lin and Li 2016). Females differ by having the spiral rod-shaped spermathecae close to the posterior margin of the epigyne, versus globular spermathecae located anteriorly in *Chanea*, as well as the copulatory ducts not being entwined with the fertilization ducts [intertwined in *Chanea* (Figs 4I, J, 6I, J, 7E–F vs. fig. 49C in Miller et al. 2009 and fig. 4C–D in Lin and Li 2016)].

###### Description.

Carapace pear-shaped, cephalic part distinctly raised in male; clypeus slightly concave. Ocular area black, AME black, others white; AER procurved, PER recurved or straight; ALE adjoined to AME and PLE, AMEs separated by at least its diameter; further separated in males than in females. Two or three pairs of strong spines on anterior surface of male chelicerae (Figs 4E, 6E). Labium fused to sternum. Sternum triangular, slightly plump, posteriorly truncated, light colour anteriorly and centrally. Each leg segment proximally pale yellow, distally darkish grey. Male with a mesal clasping spine and a distal, small nodule prolaterally on metatarsus I (Fig. 3C), female with weakly sclerotized spot on femur I. Abdomen dorsally rounded, surrounded by stripe of white pigmentation laterally and posteriorly. Venter black between epigastric furrow and spinnerets (Figs 4B, D, 6B, D, 7B).

***Male palp*.** Tibia swollen, proximally narrow and distally broad, larger number of long setae on dorsally than ventrally (Figs 3F–G, 5G–H). Cymbium translucent, encloses ventral and prolateral sides of bulb (Figs 3G, 5E, H). Paracymbium flat, wide, with a few long setae and a horn-shaped process (CyP2) distally (Figs 3D, F, 5B, D). Distal part of cymbium extends to form an apical cymbial conductor (CyC), with horn-shaped or dentoid process (CyP1) attached to lateral margin of cymbial conductor (Figs 3E, F, 5B–D, G–H). Tegulum flat, without any process or projection (Figs 3F, 5D, F). Embolus slender, filiform, elongate, encircles the bulb multiple times, end extends to apex of cymbial conductor (Figs 3F, 5E, G–H).

***Epigyne and vulva*.** Genital area covered with sparse setae, sclerotized spermathecae faintly visible through tegument (Figs 4H, 6H, 7D). Scape wrinkled, membranous, finger-like, short. Spermathecae rod-shaped, spiral, near epigynal posteromargin, separated from one another by about their length. Most of copulatory ducts membranous, extending anteriorly, coiled, overlapped with anterior end of spermathecae. Fertilization ducts relatively long, wide, originating at distal part of spermathecae, middle and proximal parts entwined with spermathecae, distal part thins gradually, inflexed (Figs 4I–J, 6I–J, 7E–F).

###### Composition.

*Chimenaqiong* sp. nov., *C.taiwanica* (Ono, 2007) comb. nov., and *C.nantou* sp. nov.

###### Distribution.

China (Hainan, Taiwan).

##### ﻿ Chimena qiong sp. nov.

Taxon classificationAnimaliaAraneaeMysmenidae

675399F5-154F-558B-AA30-83DA2C2FAC76

https://zoobank.org/B9AAA398-0B07-4F88-8477-643064500729

Figs 3 , 4 , 8

###### Type material.

***Holotype*** ♂ (**IZCAS**) and ***paratypes*** 2♀ (**IZCAS**), **China**: Hainan Province, Limushan Township, Limushan Natural Reserve, Yinhe Protected Station, 19°12.002'N, 109°43.710'E, 591±20 m, 25.III.2012, Z. Chen leg.; ***paratypes*** 2♀ (**IZCAS**), **China**: Hainan Province, Changjiang Township, Bawangling Natural Reserve, near the Yaga Convention Centre, 19°04.828'N, 109°07.369'E, 567±20 m, 13.IV.2012, Z. Chen leg.; ***Paratypes*** 1♂ (**IZCAS**); **China**: Hainan Province, Lingshui County, Diaoluoshan Natural Reserve, 18°43.505'N, 108°52.104'E, 920 m, 18.VI.2011, Y. Zhou leg.

###### Etymology.

The species epithet, a noun in apposition, refers to ‘qiong’, which is short for Hainan Province.

###### Diagnosis.

Males and females are similar to *Chimenataiwanica* comb. nov. in having a long, coiled embolus and the configuration of the vulva, but they can be distinguished by having three pairs of cheliceral spines (two pairs in the latter) (Fig. 4E vs. Fig. 6E) and a horn-shaped process (CyP1) on the cymbial conductor (tooth-shaped in the latter) (Fig. 3E, G vs. Fig. 5C, H). The female differs from congeners by the strongly spiralled, longer spermathecae (moderately spiralled in *C.taiwanica* comb. nov., and shorter in *C.nantou* sp. nov.) (Fig. 4I, J vs. Figs 6I, J, 7E, F).

###### Description.

**Male.** Habitus as in Fig. 4A, B, E, F. Total length 0.63. Carapace 0.23 long, 0.24 wide. Clypeus 0.11 high. Sternum 0.20 long, 0.19 wide. Abdomen 0.45 long, 0.44 wide. Length of legs: I 0.92 (0.29, 0.13, 0.18, 0.14, 0.18); II 0.80 (0.27, 0.09, 0.17, 0.12, 0.15); III 0.60 (0.17, 0.08, 0.12, 0.11, 0.12); IV 0.73 (0.21, 0.08, 0.15, 0.14, 0.15). Carapace pale yellow, black on cephalic area, pear-shaped. Cephalic area strongly raised. AER procurved, PER straight. Mouthparts pale brown. Chelicerae bearing 3 pairs of strong spines anteriorly (Fig. 4E). Sternum subtriangular, slightly plump, pale, anterior-centrally and laterally black, posteriorly truncated. Legs pale, gradually darkening to grey at each segment distally. Patella with distodorsal seta, proximal seta on tibia. Mesal clasping spine and distal, small nodule on metatarsus I (Figs 3C, 4B). Abdominal dorsum rounded, darkish grey, with paired light speckles, white stripe laterally and posteriorly. Posterior area of epigastric furrow and spinnerets black. Colulus black, long, tongue shaped.

***Palp*** (Fig. 3A, B, D–G): weakly sclerotized. Femur equal to 2.2× length of patella, patella approximately half of tibial width. Tibia cup-shaped, with dense, long setae dorsally. Cymbium narrow basally, wrapped around bulb ventrally and retrolaterally; distal cymbial conductor triangular, lamellar, a sub-distal tooth-shaped process (CyP1) at medial margin; paracymbium wide, earlobe shaped, bearing a few long setae and a sharp process (CyP2) distally. CyP1 almost same length as CyP2. Cymbial fold located at base of CyP1, with a few short setae (CyFs). Tegulum flat, smooth; subtegulum translucent, inner spermatic duct faintly visible. Embolus very long, filiform, tightly coiled around entire tegulum at least 10 times, distal end extending to cymbial conductor (CyC).

**Female.** Habitus as in Fig. 4C, D, G. Total length 0.72. Carapace 0.23 long, 0.25 wide. Clypeus 0.06 high. Sternum 0.19 long, 0.17 wide. Abdomen 0.48 long, 0.43 wide. Length of legs: I 0.90 (0.28, 0.12, 0.18, 0.15, 0.17); II 0.82 (0.25, 0.10, 0.18, 0.13, 0.15); III 0.63 (0.18, 0.09, 0.12, 0.11, 0.13); IV 0.76 (0.22, 0.09, 0.15, 0.14, 0.16). Cephalic area moderately raised, chelicerae unmodified, femur I with weak sclerotized spot; other features as in male.

***Epigyne*** (Fig. 4H–J): genital area bears sparse setae, with central dark speckle. Scape tongue shaped, protruded, rugose, membranous. Spermathecae long, claviform, separated by about their length, base near epigynal posterior margin. Copulatory ducts membranous, translucent, distal part overlapping and convoluted at spermathecae anteriorly. Fertilization ducts long, middle and proximal parts entwined with spermathecae, distal part extends horizontally to atrium.

###### Distribution.

**Figure 8.**
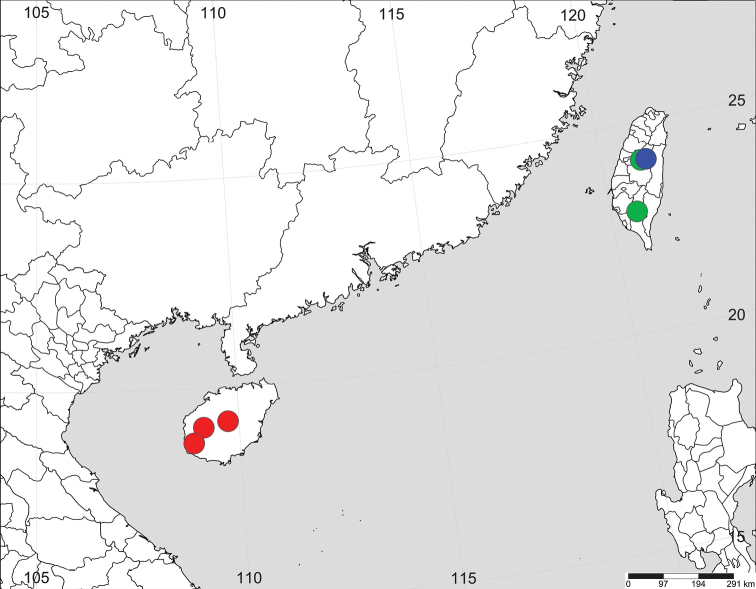
Distribution records of three *Chimena* spp.: *C.qiong* sp. nov. (red dot), *C.taiwanica* (green dot) and *C.nantou* sp. nov. (blue dot).

China (Hainan) (Fig. 8).

##### ﻿ Chimena taiwanica

Taxon classificationAnimaliaAraneaeMysmenidae

(Ono, 2007) comb. nov.

934CD32B-547E-505C-AA77-0DB2EB8D40D9

Figs 5 , 6 , 8

Mysmena taiwanica Ono, in Ono et al. 2006: 73, figs 8–19 (♂♀).

###### Type material.

***Holotype*** ♂ (**FRIT**) and ***paratypes*** 2♀ (**NSMT**), **China**: southern Taiwan, Kaohsiung Hsien, Shanping Work Station of Liukuei Research Center, ca 700 m, by sifting soil litter in a forest, 9.III.2005, H. Ono leg. Not examined.

###### Examined materials.

6♂13♀ (**IZCAS**), **China**: central Taiwan, Nantou County, Ren’ai Township, Xinsheng Village, Huisun Farm, 24°05.279'N, 121°02.078'E, 788 m, 1.VII.2013, G. Zheng leg.

###### Diagnosis.

*Chimenataiwanica* comb. nov. is similar to *C.qiong* sp. nov. in having strong, modified cheliceral spines in the males (cf. Figs 6E, 4E), a long and multi-coiled embolus (cf. Figs 5A, 3A), and in females, the similar configuration of the vulva, as in that of *C.nantou* sp. nov. (cf. Figs 6J, 4J, 7F). Males of *C.taiwanica* can be distinguished by having 2 pairs of cheliceral spines (3 pairs in *C.qiong* sp. nov.) (Fig. 6E vs. Fig. 4E and figs 8, 10 in Ono et al. 2006) and a tooth-shaped process (CyP1) (process horn-shaped in *C.qiong*) (Fig. 5C, H vs. Fig. 3E, G). The female differs from *C.qiong* sp. nov. by the moderately spiralled, thinner spermathecae tapered at the base (strongly spiralled, thicker and blunt at the base in *C.qiong* sp. nov.) (Fig. 6I, J vs. Fig. 4I, J); and from *C.nantou* sp. nov. by the longer spermathecae narrowed in the middle (shorter and wider at the middle in *C.nantou* sp. nov.) (Fig. 6I, J vs. Fig. 7E, F).

###### Description.

**Male.** Habitus as in Fig. 6A, B, E, F. Total length 0.65. Carapace 0.24 long, 0.24 wide. Clypeus 0.12 high. Sternum 0.22 long, 0.20 wide. Abdomen 0.43 long, 0.43 wide. Length of legs: I 0.96 (0.30, 0.13, 0.19, 0.14, 0.20); II 0.84 (0.27, 0.11, 0.17, 0.13, 0.16); III 0.62 (0.17, 0.09, 0.12, 0.11, 0.13); IV 0.74 (0.22, 0.09, 0.15, 0.14, 0.16). Features same as in *C.qiong* sp. nov., except for 2 paired spines on chelicerae and darker body colouration.

***Palp*** (Fig. 5A–H): weakly sclerotized. Femur equal to 2.4× length of patella, patella about half of tibial width. Tibia cup-shaped in prolateral view, slightly wider than long, bearing long setae with more dorsally than ventrally. Cymbium constricted basally, enwrapping bulb ventrally and retrolaterally; distal cymbial conductor (CyC) triangular, lamellar, tooth-shaped process (CyP1) at medial margin (Fig. 5G). Paracymbium long, with sharp distal process (CyP2), a few long setae (Fig. 5C, G). CyP1 smaller and shorter than CyP2. Cymbial fold at base of CyP1 (Fig. 5B), with a few short setae (CyFs). Tegulum flat, smooth, button-shaped (Fig. 5D); subtegulum translucent, spermatic duct faintly visible. Embolus very long, filiform, strongly sclerotized, tightly coiled around entire tegulum ca 8 times, distal end extended slightly beyond cymbial conductor (CyC) (Fig. 5G, H).

**Female.** Habitus as in Fig. 6C, D, G. Total length 0.78. Carapace 0.24 long, 0.22 wide. Clypeus 0.06 high. Sternum 0.24 long, 0.20 wide. Abdomen 0.46 long, 0.44 wide. Length of legs: I 1.00 (0.30, 0.13, 0.20, 0.15, 0.22); II 0.86 (0.27, 0.12, 0.17, 0.13, 0.18); III 0.68 (0.18, 0.09, 0.14, 0.14, 0.15); IV 0.78 (0.23, 0.10, 0.16, 0.14, 0.17). Cephalic area lower than in male, chelicerae unmodified, femur I with weak sclerotized spot; other features as in male.

***Epigyne*** (Fig. 6H–J): vulval configuration similar to *C.qiong* sp. nov. Spermathecae narrow, proximally base tapering, separated by more than their length.

###### Distribution.

China (Taiwan) (Fig. 8).

###### Remarks.

Although the type specimens of *Chimenataiwanica* comb. nov. (= *Mysmenataiwanica* Ono, 2007) have not been examined for this study, the modified strong spines on the male chelicerae, the very long, multiply coiled embolus around the bulb, the paracymbium with two processes (CyP1, CyP2), the shape of epigyne, and the protruded scape depicted in the original illustrations (see figs 8, 10, 12–15, 18–19 in Ono et al. 2006: 74–76) leave little doubt that our identification is correct. Additionally, the specimens examined here were also collected from Taiwan, not too far from the type locality.

##### ﻿ Chimena nantou sp. nov.

Taxon classificationAnimaliaAraneaeMysmenidae

407048FF-A32F-5EB0-B25F-D33540B1DE48

https://zoobank.org/DCD77110-6568-4DBA-9B03-8063C16EBC41

Figs 7 , 8

###### Type material.

***Holotype*** ♀ (**IZCAS**), **China**: Taiwan, Nantou County, Ren’ai Township, Songgang Village, 24°05.222'N, 121°10.335'E, 2067 m, 2.VII.2013, G. Zheng leg.

###### Etymology.

The new species is named after the type locality; noun in apposition.

###### Diagnosis.

*Chimenanantou* sp. nov. shares a similar configuration of the vulva to *C.qiong* sp. nov. and *C.taiwanica* comb. nov., but differs from the former by the shorter spermathecae with fewer spirals (longer and with more spirals in *C.qiong*) (cf. Fig. 7E, F vs. Fig. 4I, J), and from the latter by the more compact spermathecae (elongated in the latter) (cf. Fig. 7E, F vs. Fig. 6I, J).

###### Description.

**Female**: Habitus as in Fig. 7A–C. Total length 0.75. Carapace 0.25 long, 0.23 wide. Clypeus 0.07 high. Sternum 0.24 long, 0.22 wide. Abdomen 0.45 long, 0.42 wide. Length of legs: I 0.98 (0.30, 0.13, 0.19, 0.14, 0.22); II 0.87 (0.27, 0.12, 0.17, 0.14, 0.18); III 0.66 (0.19, 0.09, 0.14, 0.14, 0.16); IV 0.80 (0.23, 0.10, 0.16, 0.15, 0.18). Somatic features as in female of *C.taiwanica* comb. nov.

***Epigyne*** (Fig. 7D–F): vulval configuration similar to *C.qiong* sp. nov. and *C.taiwanica* comb. nov. Genital area bears sparse setae, without a dark speckle. Spermathecae short, tapering at distal end and proximal base, separated by ca 1.1× their length. Scape knob shaped, rugose, membranous. Copulatory ducts translucent. Most of fertilization ducts intertwined with spermathecae, distal part of fertilization ducts thin, inflected.

**Male.** Unknown.

###### Distribution.

China (Taiwan) (Fig. 8).

## ﻿Discussion

We tested the phylogenetic and taxonomic position of *Chimena* gen. nov. based on molecular data and unique morphological evidence. The results of our analyses indicate that *Chimena* gen. nov. is highly supported. However, further detailed phylogenetic analysis based on more mysmenid specimens will help better place the mysmenid species and genera.

## Supplementary Material

XML Treatment for Chimena

XML Treatment for Chimena qiong

XML Treatment for Chimena taiwanica

XML Treatment for Chimena nantou

## References

[B1] Baert L (1988) The Ochyroceratidae and Mysmenidae from Sulawesi (Araneae). Indo-Malayan Zoology 5: 9–22.

[B2] Brescovit AD Lopardo L (2008) The first record on the spider genus *Trogloneta* Simon in the southern hemisphere (Araneae, Mysmenidae), with descriptions of three new species from Brazil and remarks on the morphology. Acta Zoologica, Stockholm 89(2): 93–106. 10.1111/j.1463-6395.2007.00296.x

[B3] Dupérré N Tapia E (2020) Megadiverse Ecuador: A review of *Mysmenopsis* (Araneae, Mysmenidae) of Ecuador, with the description of twenty-one new kleptoparasitic spider species. Zootaxa 4761(1): 1–81. 10.11646/zootaxa.4761.1.1 33056889

[B4] Feng C Miller JA Lin Y Shu Y (2019) Further study of two Chinese cave spiders (Araneae, Mysmenidae), with description of a new genus. ZooKeys 870: 77–100. 10.3897/zookeys.870.35971 31423079 PMC6694075

[B5] Hall TA (1999) BioEdit: A user-friendly biological sequence alignment editor and analysis program for Windows 95/98/NT. Nucleic Acids Symposium Series 41: 95–98. https://10.1021/bk-1999-0734.ch008

[B6] Khmelik VV Kozub D Glazunov A (2006) Helicon Focus 3.10.3. http://www.heliconsoft.com/heliconfocus.html [accessed on 10 September 2018]

[B7] Kumar S Stecher G Li M Knyaz C Tamura K (2018) MEGA X: Molecular evolutionary genetics analysis across computing platforms. Molecular Biology and Evolution 35(6): 1547–1549. 10.1093/molbev/msy096 29722887 PMC5967553

[B8] Lanfear R Frandsen PB Wright AM Senfeld T Calcott B (2017) Partitionfinder 2: New methods for selecting partitioned models of evolution for molecular and morphological phylogenetic analyses. Molecular Biology and Evolution 34(3): 772–773. 10.1093/molbev/msw260 28013191

[B9] Li Y Lin Y (2019) Taxonomic review of the Asian *Trogloneta* species (Araneae, Mysmenidae). ZooKeys 817: 41–60. 10.3897/zookeys.817.30468 PMC6342905 30686921

[B10] Lin Y Li S (2008) Mysmenid Spiders of China (Araneae: Mysmenidae). Annales Zoologici 58(3): 487–520. 10.3161/000345408X364337

[B11] Lin Y Li S (2013) Two new species of the genera *Mysmena* and *Trogloneta* (Mysmenidae, Araneae) from southwestern China. ZooKeys 303: 33–51. 10.3897/zookeys.303.4808 PMC3689067 23794902

[B12] Lin Y Li S (2014) Mysmenidae (Arachnida, Araneae), a spider family newly recorded from Vietnam. Zootaxa 3826(1): 169–194. 10.11646/zootaxa.3826.1.5 24990042

[B13] Lin Y Li S (2016) Mysmenidae, a spider family newly recorded from Tibet (Arachnida, Araneae). ZooKeys 549: 51–69. 10.3897/zookeys.549.6046 PMC4727482 26843831

[B14] Lopardo L Giribet G Hormiga G (2011) Morphology to the rescue: Molecular data and the signal of morphological characters in combined phylogenetic analyses – a case study from mysmenid spiders (Araneae, Mysmenidae), with comments on the evolution of web architecture. Cladistics 27(3): 278–330. 10.1111/j.1096-0031.2010.00332.x 34875780

[B15] Miller JA Griswold CE Yin CM (2009) The symphytognathoid spiders of the Gaoligongshan, Yunnan, China (Araneae, Araneoidea): Systematics and diversity of micro-orbweavers. ZooKeys 11: 9–195. 10.3897/zookeys.11.160

[B16] Miller MA Pfeiffer WT Schwartz T (2010) Creating the CIPRES Science Gateway for inference of large phylogenetic trees. Gateway Computing Environments Workshop (GCE), 1–8. 10.1109/gce.2010.5676129

[B17] Ono H Chang YH Tso IM (2006) Three new spiders of the families Theridiidae and Anapidae (Araneae) from southern Taiwan. Memoirs of the National Science Museum, Tokyo 44: 71–82. 10.2476/asjaa.44.71

[B18] Ronquist F Teslenko M van der Mark P Ayres DL Darling A Höhna S Larget B Liu L Suchard MA Huelsenbeck JP (2012) MrBayes 3.2: Efficient Bayesian phylogenetic inference and model choice across a large model space. Systematic Biology 61(3): 539–542. 10.1093/sysbio/sys029 22357727 PMC3329765

[B19] Simon E (1895a) Etudes arachnologiques. 26e. XLI. Descriptions d’espèces et de genres nouveaux de l’ordre des Araneae. Annales de la Société Entomologique de France 64: 131–160.

[B20] Simon E (1895b) Histoire naturelle des araignées. Deuxième édition, tome premier. Roret, Paris, 761–1084.

[B21] Vaidya G Lohman DJ Meier R (2011) SequenceMatrix: Concatenation software for the fast assembly of multi-gene datasets with character set and codon information. Cladistics 27(2): 171–180. 10.1111/j.1096-0031.2010.00329.x 34875773

[B22] WSC (2022) World Spider Catalog. Version 23.0. Natural History Museum Bern. [accessed April 21, 2022]

